# 1633. Willingness to receive maternal RSV vaccine and infant monoclonal RSV antibody

**DOI:** 10.1093/ofid/ofad500.1467

**Published:** 2023-11-27

**Authors:** Courtney Gidengil, Jefferson M Jones, Katherine E Fleming-Dutra, Jamison Pike, Mila Prill, Patricia Wodi, Megan Lindley, Amber Gedlinske, Andrew Parker, Aaron Scherer

**Affiliations:** RAND Corporation, Boston, Massachusetts; CDC, Atlanta, Georgia; Centers for Disease Control and Prevention, Atlanta, Georgia; Centers for Disease Control and Prevention (CDC), Atlanta, Georgia; Centers for Disease Control and Prevention, Atlanta, Georgia; Centers for Disease Control and Prevention, Atlanta, Georgia; Centers for Disease Control & Prevention, Atlanta, GA; University of Iowa, Iowa City, Iowa; RAND Corporation, Boston, Massachusetts; University of Iowa, Iowa City, Iowa

## Abstract

**Background:**

RSV results in substantial morbidity among young infants. Maternal RSV vaccines and monoclonal antibodies against RSV for infants have been developed, but little is known about acceptance of these products.

**Methods:**

We conducted an online survey of U.S. adults who were pregnant (50%) or < 12 months postpartum (50%) between December 21, 2022 and January 2, 2023. Primary outcomes in two logistic regression models were willingness to receive RSV vaccine during pregnancy (definitely or probably would) and willingness to give their infant RSV monoclonal antibody. Covariates included prior vaccinations, vaccine attitudes, RSV risk perceptions, and RSV knowledge.

**Results:**

Among respondents (N=523), mean age was 32 years (SD + 8 years); 48% had a high school education or less. In the last 12 months, 56% had received a COVID-19 vaccine and 58% an influenza vaccine; 66% had received Tdap during the pregnancy or intended to. A third of respondents thought their infant would get RSV in the first year of life; 14% thought severe illness would result. Sixty-one percent were willing to receive maternal RSV vaccine and 70% were willing for their infant to receive monoclonal antibody; 74% expressed willingness for at least one of these products. Predictors of maternal RSV vaccine willingness were Tdap during pregnancy (adjusted Odds Ratio [aOR] 2.83; 95% CI 1.75, 4.59), influenza vaccine in last 12 months (aOR 1.63; 95% CI 1.01, 2.63), positive feelings towards vaccines (aOR 4.15; 95% CI 2.49, 6.91), and higher perceived likelihood of their infant getting RSV illness (aOR 2.42; 95% CI 1.43, 4.09). Predictors of infant monoclonal antibody willingness were Tdap during pregnancy (aOR 2.50; 95% CI 1.52, 4.11), positive feelings towards vaccines (aOR 2.52; 95% CI 1.49, 4.26), and higher perceived likelihood of RSV illness (aOR 3.57; 95% CI 1.95, 6.53); living in the South versus Northeast was associated with lower willingness (aOR 0.41; 95% CI 0.20, 0.84).

**Figure:**
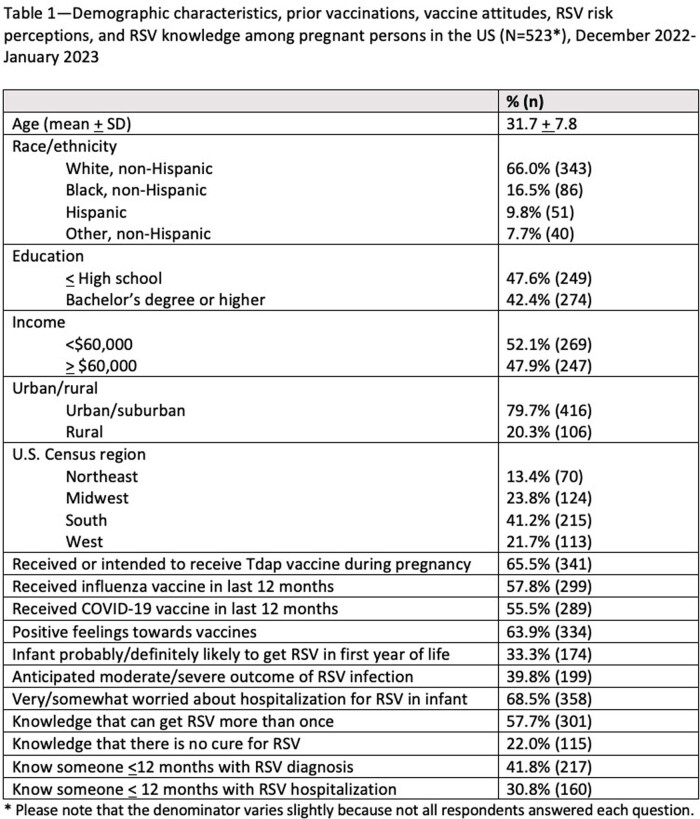

**Figure:**
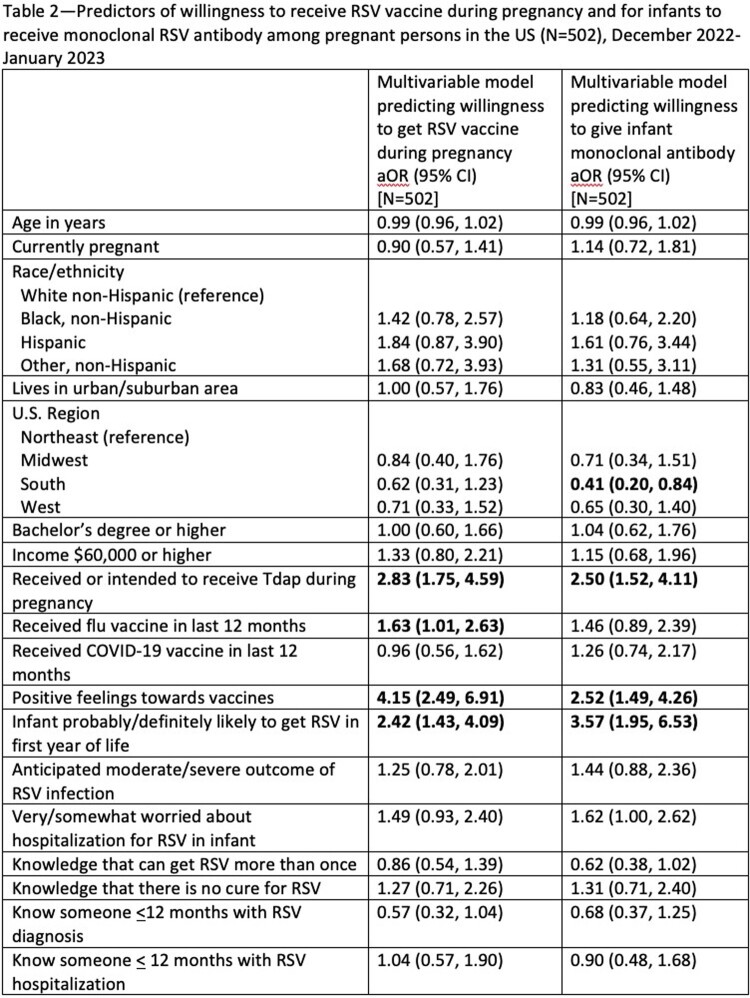

**Conclusion:**

Most respondents (74%) were willing to receive RSV vaccine and/or give their infant RSV monoclonal antibody. Receiving Tdap during pregnancy, favorable attitudes to vaccines, and higher perceived likelihood of RSV illness were associated with willingness for these products, which may inform future roll-out.

**Disclosures:**

**All Authors**: No reported disclosures

